# Minimally Invasive Esthetic Treatments With an Orthorestorative Integrated Digital Approach: A Case Report

**DOI:** 10.1155/crid/1779076

**Published:** 2025-11-26

**Authors:** Camillo D'Arcangelo, Matteo Buonvivere, Mirco Vadini, Francesco De Angelis

**Affiliations:** Department of Medical, Oral and Biotechnological Science, School of Dentistry, “G. D'Annunzio” University, Chieti-Pescara, Italy

**Keywords:** case report, dental esthetics, digital technology, gingivectomy, lithia disilicate, removable orthodontic appliances, workflow

## Abstract

The article describes the effectiveness of an integrated digital approach combining orthodontic and restorative phases in dental esthetic treatment, focusing on minimally invasive procedures. A 55-year-old woman with dental esthetic concerns underwent a comprehensive treatment plan. Initial evaluation included digital impressions of both arches using an intraoral scanner, followed by a 15-week sectional clear aligner therapy to correct dental misalignments prior to minimally invasive lithium disilicate veneers. A digital wax-up was proposed to the patient and used as a guide to perform gingivectomy and minimal tooth reduction. The aligner therapy effectively corrected the misalignment and crossbite, optimizing tooth positioning for minimally invasive veneer preparation. Digital planning allowed precise enamel reduction and gingival reshaping. The final placement of lithium disilicate veneers resulted in significant esthetic improvement, confirmed by clinical evaluations and patient satisfaction. The integration of digital technologies in orthodontic and restorative dentistry enhances esthetic treatment planning and execution. Combining sectional clear aligner therapy with precise digital wax-ups minimizes invasive procedures, improves surgical precision, and ensures predictable outcomes. The digital workflow also facilitates patient education and communication, demonstrating treatment goals and results effectively.

## 1. Introduction

In the modern beauty-conscious society, patients are increasingly aware of the dental esthetics impact [1, 2]. With the progressive decrease of dental caries [3], they often address the dentist with concerns regarding the alignment and color of their upper anterior teeth, without any dental health issue [4]. In a similar patient, any restorative treatment should be as respectful as possible of the sound tooth structure, which should ideally be left untouched. Specifically, when veneers are needed in case of crowding/misalignment, an orthodontic treatment with braces or aligners should precede the prosthetic phase in order to straighten the teeth and minimize their preparation [5, 6]. On this basis, an integrated digital approach combining the orthodontic and the restorative phases in a single platform could be a valid aid for the clinician. From the tooth alignment to the digital wax-up upon which to base targeted tooth reduction and even surgical reduction guides, each step could in fact be carefully planned, visualized, and shown to the patient. Such an approach is illustrated in the following case report.

## 2. Case Presentation

**Figure 1:**
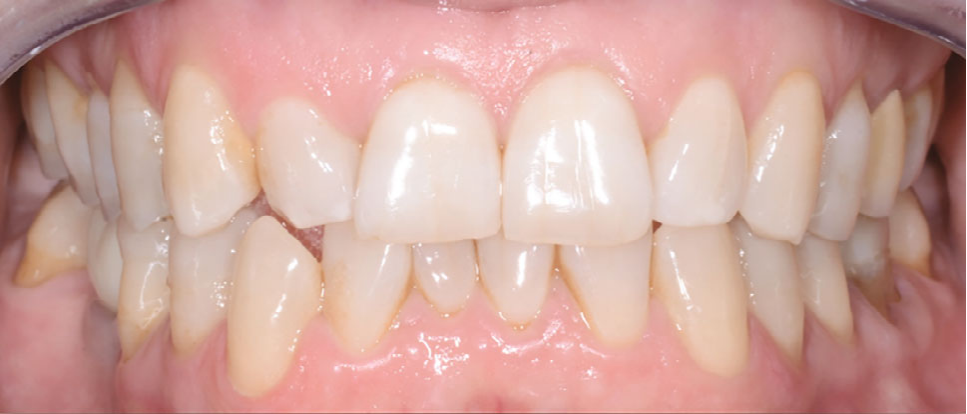
Intraoral photo showing a dental lower midline deviation, a lower right canine crossbite, and mild crowding of the lower anterior sextant.

A 55-year-old woman was referred to the dental clinic with the chief complaint of unsatisfactory smile appearance. The patient's medical and dental history was unremarkable. The intraoral inspection revealed a dental lower midline deviation, a lower right canine crossbite, and mild crowding of the lower anterior sextant. Lower incisors also showed signs of wear (Figure 1).

The clinical examination excluded dysfunctions affecting the temporomandibular joint, the masticatory muscles, and the associated structures. No symptoms of temporomandibular disorders were reported by the patient. The proposed and accepted treatment plan consisted of canine crossbite and crowding correction with clear sectional aligners, followed by additive lithium disilicate veneers on the upper anterior teeth, from the left second premolar to the right one.

**Figure 2:**
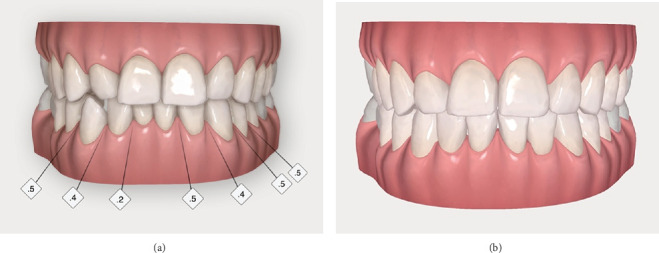
(a, b) ClinCheck 3D software platform showing the sectional clear aligner therapy details and results.

After standard oral hygiene procedures, a digital impression of both the upper and lower arches was taken by means of an intraoral scanner (iTero Lumina, Align Technology Inc., San Jose, California, United States) and sent to the manufacturer in order to obtain the tridimensional simulation of the tooth movements through the ClinCheck 3D software platform (Align Technology Inc.). A 15-week sectional clear aligner therapy (Invisalign GO, Align Technology Inc.) aimed at correcting the right lower canine crossbite and the lower anterior crowding was proposed, thus leaving the upper anterior teeth in the ideal position to minimize the tooth preparation for veneers (Figure 2).

**Figure 3:**
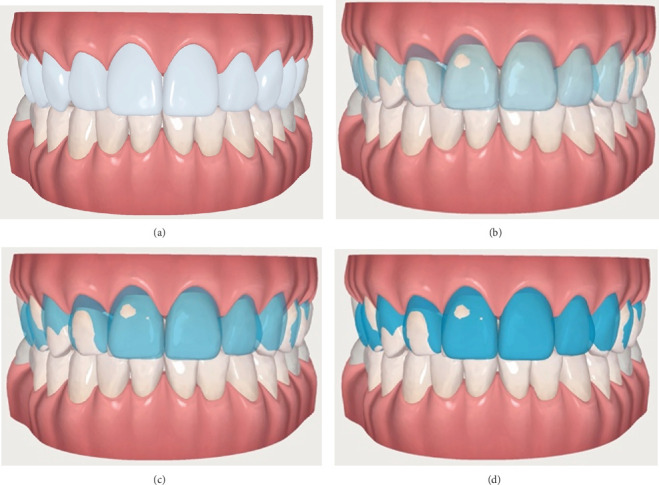
Smile Architect digital wax-up (a) superimposed on the ClinCheck at crescent opacity levels (b–d).

A digital wax-up was then produced based on the same ClinCheck with the Smile Architect software (Align Technology Inc.) (Figure 3a). The Smile Architect software automatically generated the most suitable wax-up for the patient, based on the conventional digital smile design principles, which include a careful analysis of incisal edge position, dental proportions, and facial references [7]; minimal corrections were manually implemented by the clinicians, in order to further improve the automatically generated digital wax-up. Using the “Restor” feature of the software, the proposed wax-up was then superimposed at different opacity levels on the ClinCheck (Figures 3b, 3c, and 3d) to evaluate the future veneer thickness (and therefore the need for tooth reduction) and their relationship with the gum line. Specifically, working with the “Mass” feature of the software, it was possible to evaluate the exact location and amount of enamel to be reduced, ranging from 0.5 mm on the buccal surface of the upper right lateral incisors to 1–1.5 mm on the tip of the upper right canine, while the vast majority of enamel could be left untouched. The whole digital planification was shown and carefully explained to the patient, who gave his written informed consent and accepted the treatment plan.

The treatment started with the sectional clear alignment phase. Interproximal reduction was performed following the producer's recommendations: 0.5 mm of enamel was removed between 4.3 and 4.4, 0.4 mm between 4.2 and 4.3, 0.2 mm between 4.1 and 4.2, 0.5 mm between 3.1 and 3.2, 0.4 mm between 3.2 and 3.3, 0.5 mm between 3.3 and 3.4, and 0.5 mm between 3.4 and 3.5 (Figure 2). The whole therapy required 15 aligners. The patient was instructed to wear them for at least 22 h a day and to switch to a new aligner each week. Clinical evaluations were conducted every 4 weeks throughout the treatment. At the end of the therapy, the anterior tooth misalignment was corrected, as well as the lower right canine crossbite, thus obtaining an appropriate overjet to safely restore the upper anterior teeth.

**Figure 4:**
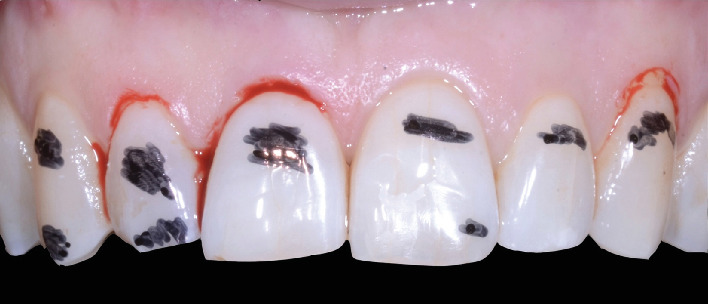
Clinical photo showing the result of the minimally invasive gingivectomy performed on Teeth 1.1, 1.2, and 2.3. The areas marked in black needed to be reduced according to selective area reduction.

**Figure 5:**
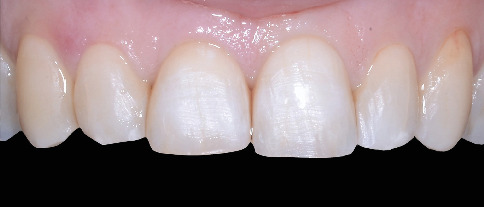
Clinical photo showing the result of selective area reduction.

After the alignment, the digital wax-up was 3D-printed (Pro S 3D Printer, SprintRay, Los Angeles, California, United States) and used to fabricate two acrylic resin templates: the first one to guide the gum line reshaping and the second one to mark on the teeth to be prepared the exact areas to reduce, thus performing an extremely targeted enamel preparation named selective area reduction (SAR) [8]. Using the templates, this information was transferred into the patient's mouth and a minimally invasive gingivectomy was performed on Teeth 1.1, 1.2, and 2.3 (Figure 4) prior to SAR (Figure 5). Subsequently, #000 retraction cord (Ultrapak, Ultradent Products Inc., South Jordan, Utah, United States) was placed in the sulcus of each prepared tooth; a new impression was taken with the intraoral scanner (iTero Lumina, Align Technology Inc.) and sent to the laboratory for the six lithium disilicate veneer fabrication (e.max Press HT, Ivoclar Vivadent Inc., Schaan, Liechtenstein). After the try-in, the veneers were etched using 5% hydrofluoric acid and silanized. Subsequently, standard adhesive procedures were performed under rubber dam isolation: Each tooth was sandblasted with aluminum oxide particles and then etched with 37% orthophosphoric acid followed by thorough water rinse. Each tooth was then coated with a layer of adhesive (Enabond, Micerium S.p.A., Avegno, Genoa, Italy) left unpolymerized prior to the veneer luting with prewarmed composite (Enamel Plus Hri Function, Micerium S.p.A.). After gross luting excess removal, a short initial light-curing of 5 s per veneer was performed and any other interproximal or cervical flash material was carefully removed with manual instruments under high magnification. Rotary instruments were avoided to preserve the veneers' marginal fit and glossy appearance obtained with laboratory polishing procedures. Finally, a complete light polymerization cycle of two 40-s sessions was performed on the oral and buccal side of each veneer under glycerin gel.

**Figure 6:**
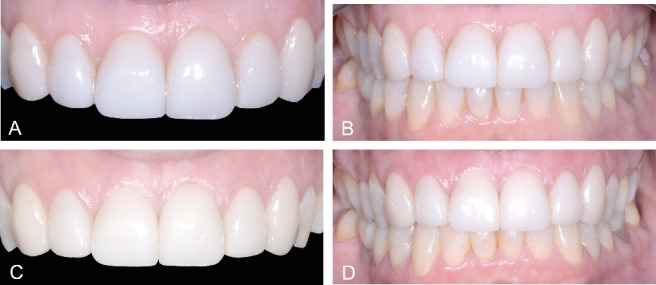
Details of the lithium disilicate veneers (A) and final esthetic result (B) after 1 week and at the 36-month follow-up (C, D).

The clinical postoperative photograph taken after 1 week showed proper gum healing and satisfactory results (Figure 6a,b), with no detectable visible plaque and bleeding on probing on the restored teeth. Adequate esthetics and periodontal health were effectively maintained at the 36-month follow-up (Figure 6c,d).

## 3. Discussion

Patients' esthetic perception revealed to be different from the dentists' one [9], being mainly influenced by tooth color, misalignment, crowding, and gingival display [10, 11]. In this light, patients' esthetic preferences have to be considered to create a tailor-made treatment. A planning software integrating tooth alignment and restorative design in a single platform could be a valid aid in treatment plan communication and patients' acceptance [12]. After a first visit including a digital impression, in fact, dentists could show patients not only a timed visual representation of tooth movement and straightening but also a quite accurate image of the prosthetic results. Specifically, superimposing the wax-up on the ClinCheck, the patient could visually understand the need for enamel reduction and gingivectomy. Moreover, the possibility to add a facial rendering of the patient allows creating facially driven orthorestorative treatment plans visually involving the patient itself, who will therefore receive unmatched education and motivation.

Despite its communicative power, a similar digital approach also holds practical value for the clinician. Since a gingival architecture in harmony with tooth morphology is paramount for the overall smile perception, restorative treatments often require a preliminary gingivectomy [13]. In the past, gingivectomy was traditionally performed freehand, thus relying solely on the surgeon's experience and visual evaluation. Recently, several techniques have been proposed to fabricate vacuum-formed surgical guides based on the technician's wax-up. Despite helping the clinician to perform more precise and predictable surgery [13], however, vacuum-formed guides showed several disadvantages, such as the need to be produced on a cast and waxed model (thus requiring a traditional workflow) or an excessive thickness [14]. These limitations could be overcome by digitally designed 3D-printed surgical guides like the one described in the present case.

In the era of minimally invasive care, not only dental professionals, but even patients recognize the importance of preserving sound dental tissues, especially when treatments are performed solely for esthetic purposes. When planning for veneers, a no-prep or minimal-prep approach should therefore be mandatory [15]. A targeted preparation strategy like the SAR, in fact, shows several advantages in terms of both clinical procedures and long-term results. From a clinical point of view, the SAR could be performed without anesthetic and eases the impression-taking phase, since no thin cervical enamel margin has to be recorded [15]. Moreover, since natural teeth are barely touched, there is no need for intermediate provisional restorations, whose placement in an area at high esthetic demand requires skill by both the clinician and the technician [15]. In terms of long-term results, a minimal-prep approach likely saves an abundant amount of enamel. On the one hand, this increases the bond strength and marginal integrity of the ultrathin veneers [15–17]. On the other hand, saving enamel allows noninvasive reintervention possibly needed in the long run.

Finally, a full digital approach simplifies the technician's work. Lithium disilicate restorations, in fact, could be designed and produced in a very quick way, without the need to switch to an analog workflow relying on multiple gypsum models. In this way, several operator-dependent steps slowing down the process and potentially increasing inaccuracy could be avoided [18]. Moreover, having taken into account the patient's feedback in each step of the planning phase, the need for modifications after the try-in is considerably reduced, thus streamlining the laboratory procedures. Despite several mechanical advantages [19, 20], monolithic lithium disilicate restorations could present some esthetic limitations. Compared to stratified ceramics, monolithic lithium disilicate lacks the usual incisal third effects such as well-defined mamelons, opalescence, and white spots. However, pleasant esthetic results could still be achieved by means of superficial staining, which represents a quick and cost-effective alternative to full-thickness or partial ceramic veneering [21].

The integration of digital technologies in orthodontic and restorative dentistry offers significant advantages in esthetic treatment planning and execution. By combining clear aligner therapy with precise digital wax-ups generated by the same software, clinicians can optimize tooth alignment and minimize the need for invasive tooth preparation. The use of guides based on 3D-printed wax-ups enhances surgical precision during gingivectomy and invasiveness during SAR, thus contributing to predictable outcomes and patient satisfaction. Moreover, the digital workflow facilitates patient education and communication by visually demonstrating treatment goals and anticipated results.

## Data Availability

The data that support the findings of this study are available from the corresponding author upon reasonable request.
